# Ahmed Glaucoma Valve with Adjunctive Amniotic Membrane for Refractory Glaucoma

**Published:** 2010-07

**Authors:** Heydar Amini, Mohammad yaser Kiarudi, Sasan Moghimi, Ghasem Fakhraie, Nima Amini

**Affiliations:** Farabi Eye Research Center, Department of Ophthalmology, Tehran University of Medical Sciences, Tehran, Iran

**Keywords:** Shunt, Amniotic Membrane, Refractory Glaucoma, Glaucoma Drainage Device

## Abstract

**Purpose:**

To evaluate the efficacy and safety of Ahmed Glaucoma Valve (AGV) implantation with adjunctive use of preserved amniotic membrane for surgical management of refractory glaucoma.

**Methods:**

Seven patients (5 female subjects) with refractory glaucoma were included in the study. An AGV (model FP7) was implanted in the usual manner and was covered with two layers of cryopreserved human amniotic membrane. Intraocular pressure (IOP) and number of glaucoma medications before and after surgery, and complications were evaluated.

**Results:**

Mean duration of follow-up was 16.8±4.6 months. Mean preoperative IOP was 31.7±4.4 mmHg which was reduced to 17.7±6.1 mmHg at final follow-up (P=0.01, Wilcoxon U test). Although the number of topical medications was also reduced (mean decrease of 0.85 drops), this decrease was not significant (P=0.10, Wilcoxon U test). None of the eyes developed encapsulation after surgery; only one case was complicated by posterior migration of the implant resulting in failure.

**Conclusion:**

Glaucoma shunt surgery using the AGV with adjunctive amniotic membrane seems to be a safe and effective procedure which may reduce the risk of bleb encapsulation in refractory glaucomas.

## INTRODUCTION

Glaucoma drainage devices (GDDs) and cyclodestructive procedures are the usual surgical options in patients with multiple previous glaucoma operations.^1^ Implanting GDDs in eyes with scarred or thin conjunctiva due to prior surgical procedures is difficult and may entail serious complications with unfavourable long-term results.^2^ Amniotic membrane (AM) has anti-inflammatory, antifibrotic, and antiangiogenic properties^3^, and can improve epithelialization of the ocular surface and act as an inhibitor of fibrosis.^4^ These features theoretically make the AM an ideal adjuvant in trabeculectomy and shunting procedures to reduce fibrosis. Recently, there have been promising results with application of AM during trabeculectomy in both animal^5^ and human subjects^6^–^8^. In an experimental study, Barton et al^5^ examined conjunctival specimens from 24 albino rabbits that had undergone glaucoma filtration surgery with AM. Compared with unoperated conjunctiva, significantly less fibroblast outgrowth was found in tissue cultures of AM transplantation explants.

A review of the literature shows that the application of AM during shunting procedures is still obscure. In this study, we introduce the adjunctive use of amniotic membrane for GDD implantation and report its safety and efficacy in refractory glaucomas.

## METHODS

Patients with refractory glaucoma in whom previous trabeculectomy procedures had failed were included in this study. All patients had intraocular pressure (IOP) exceeding 22 mmHg with maximally tolerated medications. Exclusion criteria were previous glaucoma shunt surgery, previous scleral buckling, and anterior staphyloma in the superotemporal quadrant. All subjects underwent implantation of an Ahmed Glaucoma Valve (AGV) model FP7 (New World Medical Inc., Rancho Cucamonga, USA) through a fornix-based incision in the superotemporal quadrant. The implant was fixed 9 to 10 mm posterior to the surgical limbus with two 9-0 nylon sutures. The tube was then inserted into the anterior chamber through a 23-gauge needle track. After covering the tube with a scleral patch graft, the shunt plate was covered with two layers of cryopreserved human amniotic membrane, stromal side down, without any sutures. The conjunctiva was repaired using 10-0 nylon sutures.

All patients were visited one day, 1 week, and 1, 3 and 6 months after the procedure, and every 6 months thereafter. Pre- and postoperative IOP, the number of topical medications before and after surgery, and complications were recorded. Surgical success was defined as IOP of 5 to 22 mmHg with or without use of medications. Failure was defined as IOP higher than 22 mmHg with medications or an IOP lower than 5 mmHg on two consecutive visits, or signs of hypotony maculopathy. Bleb encapsulation was diagnosed in the presence of increased IOP, patency of the tube (documented by an elevated bleb), and slit-lamp confirmation of fibrous encapsulation over the shunt plate at least 1 month after surgery.

Descriptive statistics were used to report demographic characteristics using the SPSS software package version 14.5 (SPSS Inc., Chicago, USA). Wilcoxon U test was used for comparing variables before and after surgery.

## RESULTS

Seven patients (including 5 female subjects) with mean age of 18.4±18.3 (range, 1–50) years were operated and followed for 16.8±4.6 (range, 9–22) months (Table 1). Mean preoperative IOP was 31.7±4.4 mmHg which was reduced to 17.7±6.1 mmHg at final follow-up (P=0.01, Wilcoxon U test). There was a decrease in the mean number of topical medications from 2.42 preoperatively to 1.57 after surgery (mean decrease of 0.85 drops). However, this reduction was not statistically significant (P=0.10, Wilcoxon U test). No case of encapsulation was observed during the follow-up period. Only one eye was complicated by posterior migration of the implant which resulted in shunt failure (success rate, 85.6%). Another minor complication was a small Descemet membrane detachment which required no intervention.

## DISCUSSION

The history of aqueous shunts dates back to more than 100 years ago with the use of a range of materials to accomplish artificial translimbal or transscleral drainage of aqueous humor.^9^,^10^

Amniotic membrane is now used in a wide variety of medical conditions. The membrane contains a host of growth factors, anti-inflammatory cytokines and antiangiogenic factors which promote epithelial cell proliferation and wound healing, and at the same time suppress inflammation and neovascularizaton.^10^,^11^

There are several reports on the efficacy of AM application during trabeculectomy.^5^–^8^ Sheha et al^8^ showed that in refractory glaucoma, trabeculectomy combined with mitomycin C (MMC) and amniotic membrane transplantation had higher success rates, lower mean postoperative IOPs, and lower complication rates compared to trabeculectomy with MMC alone. Drolsum and associates^1^ evaluated the results of amnion-shielded trabeculectomy with concomitant use of mitomycin C. They suggested this procedure as an option in cases with previously failed filtering surgery with thin or scarred conjunctiva.

The benefit of antifibrotic agents used as adjuncts to GDDs is controversial. According to the Cochrane Review on aqueous shunts^12^, among three randomized controlled clinical trials, two concluded that antifibrotic agents (e.g., MMC) have no beneficial long-term effect when used with aqueous shunts. Only Duan et al^13^ concluded that adjunctive MMC used with the Hunan aqueous device is associated with better success rates. However, as noted in the Cochrane Review, this study suffers from several methodological flaws. Minckler et al^10^ stated that there is no benefit in using antifibrotic agents as adjuncts to aqueous shunt procedures^10^.

The seven patients included in the current report were all high-risk surgical candidates in whom previous procedures had failed and all except one, showed considerable IOP reduction and a decrease in the number of glaucoma medications. The success rate of 85.6% is comparable to the highest success rates reported for AGV implants.^14^–^16^ The cumulative probability of success was 87% at 1 year and 75% at 2 years in the study by Huang et al^14^; 87% at 1 year and 82% at 2 years in the report by Topouzis et al^15^; and 82.9% at 1 year in the study by Tsai et al^16^. The success rate observed in our results may be attributed to addition of amniotic membrane to standard shunting procedure.

Some degree of fibrous encapsulation is expected to develop around most GDDs. This encapsulation is more severe and has an earlier onset with the AGV implant as opposed to Baerveldt or Molteno shunts^16^ in which filtration is delayed due to tube ligation; this may reflect the effect of immediate aqueous filtration on fibrous encapsulation.

Bleb encapsulation, months after surgery, is particularly frustrating and rarely responds to needling (with or without fluorouracil injections) or surgical bleb revision with antimetabolites. Frequently, an additional glaucoma procedure, such as implanting another drainage device inferonasally, or laser cyclodestruction is necessary.^2^,^9^,^16^ Tsai et al^16^ investigated the outcomes of Baerveldt and Ahmed implants for treatment of refractory glaucoma. Complications associated with both implants included choroidal effusions, clinical bleb encapsulation, and other postoperative complications resulting in surgical failure such as suprachoroidal hemorrhage, tractional retinal detachment, endophthalmitis, and malignant glaucoma. The Ahmed implant had a higher incidence of encapsulation (29 of 48 patients; 60.4%) and earlier mean time to initial observation of encapsulation (50.0±43.8 days)^16^. None of the patients in our study developed bleb encapsulation during a mean follow-up period of 16.8 months. Similar to our results, were those of Eliezer et al^4^ who compared the safety and efficacy of AM in trabeculectomy for treatment of primary open-angle glaucoma. They, too, reported an encapsulated bleb in one eye in the AM group versus 3 eyes in the control group. These observations may be due to the fact that AM contains a host of growth factors, anti-inflammatory cytokines, and antiangiogenic factors which promote epithelial cell proliferation and wound healing, and at the same time suppress inflammation and neovascularization.^12^

One limitation of our study is the small sample size. Another might be that there was a wide age range of glaucoma types and patient age. Although the duration of follow-up was relatively long, these limitations make it difficult to draw a definite conclusion. Considering the results of this study and promising results of trabeculectomy with AM transplantation^1^,^6^–^8^, AGV implantation with amniotic membrane transplantation can be considered as an option for surgical treatment of refractory glaucomas. However, these preliminary findings should be confirmed by future investigations.

## Figures and Tables

**Table 1:** Characteristics of patients undergoing Ahmed glaucoma valve implantation with amniotic membrane

	Age (years)	Sex	Type of Glaucoma	OD/OS	History of Surgery	Pre-op BCVA	Pre-op IOP	Pre-op No. of Medications	F/U (months)	Post-op BCVA	Post-op IOP	Post-op No. of Medications	Position of shunt	Complication
1	1	M	Congenital	OD	Trabeculotomy, Trabeculectomy	-	28	3	18	-	26	2	under conjunctiva	Posterior migration of implant
2	16	F	Juvenile	OS	Twice Trabeculectomy	20/200	30	2	19	20/60	18	2	good	Descemet detachment
3	6	F	Aphakic	OD	Lensectomy Trabeculectomy	-	34	3	18	20/200	20	2	good	-
4	36	F	Secondary (traumatic)	OD	Repaired Corneal Laceration, Lensectomy, PK	20/200	26	3	9	20/400	20	-	good	-
5	17	F	Congenital	OD	Trabeculotomy, Trabeculectomy	20/30	38	2	22	20/30	19	3	good	-
6	50	F	CACG	OS	Cataract Extraction, Twice Trabeculectomy	20/60	36	2	20	20/60	15	2	tube-iris touch	-
7	3	M	Congenital	OD	Trabeculotomy, Trabeculectomy	-	30	2	12		6	-	good	-

OD, right eye; OS, left eye; Pre-op, preoperative; BCVA, best corrected visual acuity; IOP, intraocular pressure; F/U, follow-up; Postop, postoperative; M, male; F, female; CACG, chronic angle closure glaucoma; PK, penetrating keratoplasty
